# Cadmium and Proliferation in Human Uterine Leiomyoma Cells: Evidence of a Role for EGFR/MAPK Pathways but Not Classical Estrogen Receptor Pathways

**DOI:** 10.1289/ehp.1408234

**Published:** 2014-10-24

**Authors:** Xiaohua Gao, Linda Yu, Alicia B. Moore, Grace E. Kissling, Michael P. Waalkes, Darlene Dixon

**Affiliations:** 1 Molecular Pathogenesis Group, National Toxicology Program (NTP) Laboratory, Division of the NTP (DNTP); 2 Biostatistics Branch, Division of Intramural Research (DIR) and DNTP, and; 3 Inorganic Toxicology Group, DNTP, National Institute of Environmental Health Sciences, National Institutes of Health, Department of Health and Human Services, Research Triangle Park, North Carolina, USA

## Abstract

**Background:**

It has been proposed that cadmium (Cd) is an environmental “metalloestrogen” and that its action is mediated via the estrogen receptor (ER). Cd mimics the effects of estrogen in the rat uterus, and blood Cd concentrations positively correlate with ER levels in uteri of women with fibroids.

**Objectives:**

In the present study we explored whether Cd could stimulate proliferation of estrogen-responsive human uterine leiomyoma (ht-UtLM) cells and uterine smooth muscle cells (ht-UtSMCs) through classical interactions with ERα and ERβ, or by nongenomic mechanisms.

**Methods:**

We used estrogen response element (ERE) reporters, phosphorylated receptor tyrosine kinase arrays, Western blot analysis, estrogen binding, and cell proliferation assays to evaluate the effects of Cd on ht-UtLM cells and ht-UtSMCs.

**Results:**

Cd stimulated growth of both cell types at lower concentrations and inhibited growth at higher concentrations (≥ 50 μM). Cd did not significantly bind to ERα or ERβ, nor did it show transactivation in both cell types transiently transfected with ERE reporter genes. However, in both cells types, Cd (0.1 μM and 10 μM) activated p44/42 MAPK (ERK1/2), and a MAPK inhibitor (PD98059) abrogated Cd-induced cell proliferation. Cd in ht-UtLM cells, but not in ht-UtSMCs, activated the growth factor receptors EGFR, HGFR, and VEGF-R1 upstream of MAPK. Additional studies in ht-UtLM cells showed that AG1478, an EGFR inhibitor, abolished Cd-induced phosphorylation of EGFR and MAPK.

**Conclusions:**

Our results show that low concentrations of Cd stimulated cell proliferation in estrogen-responsive uterine cells by nongenomic activation of MAPK, but not through classical ER-mediated pathways.

**Citation:**

Gao X, Yu L, Moore AB, Kissling GE, Waalkes MP, Dixon D. 2015. Cadmium and proliferation in human uterine leiomyoma cells: evidence of a role for EGFR/MAPK pathways but not classical estrogen receptor pathways. Environ Health Perspect 123:331–336; http://dx.doi.org/10.1289/ehp.1408234

## Introduction

Cadmium (Cd) is a toxic metal and common environmental contaminant, with human exposures most commonly occurring through occupational inhalation, tobacco use, ingestion (food and drinking water), or inhalation of ambient air (Agency for Toxic Substances and Disease Registry 2012; International Agency for Research on Cancer 2012). Data from the National Health and Nutrition Examination Survey (NHANES 2011) show that > 60% of the U.S. population has detectable blood Cd levels (range, 1.25–77.14 nmol/L). Chronic Cd exposure has been associated with increased lung and prostate cancers in occupationally exposed workers in the United States (Verougstraete et al. 2003), and elevated levels of serum Cd correlate with human pancreatic cancer (Kriegel et al. 2006). Evidence from rodent and *in vitro* studies shows a direct causal link between Cd and cancer (Jing et al. 2012; Qu et al. 2012). Molecular studies have suggested that the underlying mechanism of the carcinogenic activity of Cd is multifactorial and may include DNA damage (Zhang et al. 2010), phenotype transitioning (Benbrahim-Tallaa et al. 2009), modification of Cyp1a1 (cytochrome P450) expression (Kluxen et al. 2012), Sp1 inactivation (Youn et al. 2005), and promotion of angiogenesis (Jing et al. 2012).

Recent studies have suggested that Cd is an environmental “metalloestrogen” with effects that may be mediated by the estrogen receptor (ER) (Johnson et al. 2003; Kluxen et al. 2012). The proposition of Cd being an endocrine disruptor is plausible due to reports of its wide spectrum of deleterious effects on experimental *Xenopus laevis* (Lienesch et al. 2000), mice (Ali et al. 2010), rats (Johnson et al. 2003), and the developing human reproductive tract (Kippler et al. 2012). Johnson et al. (2003) reported that exposure of ovariectomized rats to Cd resulted in increased uterine wet weight with accompanying proliferation of the endometrium and induction of the progesterone receptor. Other investigators have reported that Cd regulates progesterone synthesis in cultured granulosa cells (Nampoothiri et al. 2007) and in pseudo-pregnant rats (Henson and Chedrese 2004). In addition, a recent cohort study suggested a definite role for Cd in postmenopausausal breast cancer in women (Julin et al. 2012), which is consistent with prior observations of Cd’s ability to transform human breast epithelial cells into a cancer phenotype *in vitro* (Benbrahim-Tallaa et al. 2009).

There is reasonable evidence suggesting that Cd may be associated with uterine disease in women (Jackson et al. 2008). Nasiadek et al. (2005) detected Cd in uterine tissue of women with leiomyoma, although the concentrations were slightly lower than in surrounding myometrium. These investigators also found that tissue Cd levels correlated with levels of ER expression in leiomyoma tumors, indicating a possible link between Cd and estrogen signals (Nasiadek et al. 2011). Considering that uterine fibroids (i.e., leiomyomas, myomas) are one of the most common hormonally responsive tumors clinically affecting women of reproductive age, it is a first-line strategy to identify potential environmental risk factors for the management of this disease (Di et al. 2008; Gao et al. 2012).

The ability to activate ERα is central to estrogen and “estrogen mimics” inducing cell proliferation in many cancers and other disease processes (Osborne and Schiff 2005). At the molecular level, estrogens, such as 17β-estradiol (E_2_), bind to either ERα or ERβ and function through classical or nongenomic signaling pathways, with the latter including the pro-proliferation, mitogen-activated protein kinase (MAPK)/ERK1/2 signaling pathway (Creighton et al. 2006). The MAPK pathway is a critical regulator of cell proliferation in both normal development and tumor growth. (Dhillon et al. 2007; Osborne and Schiff 2005). Conversely, the role of ERβ has largely been associated with inhibition of proliferation or proapoptotic events when coexpressed with ERα; however, recent studies in ERα-negative breast cancer cells may suggest a role of ERβ in cell survival (Leygue and Murphy 2013).

In the present study, we examined whether Cd could induce growth in estrogen-responsive human uterine fibroid and myometrial cells, and if so, did ERs mediate the effects. We first examined the effects of low and high concentrations of Cd on cell growth, and then explored possible molecular mechanisms mediating any Cd-induced effects. Our results have important clinical and environmental risk implications, and provide evidence of a molecular mechanism of Cd-induced effects in uterine fibroid cells.

## Materials and Methods

*Cells and reagents*. The UtLM-hTERT (ht-UtLM) cells and UtSMC-hTERT cells (ht-UtSMCs) (passage 24) were established in our laboratory and maintained in supplemented medium as previously described (Carney et al. 2002). Cadmium chloride (CdCl_2_; 99.999%, catalog no. 439800; Sigma-Aldrich) was dissolved in double distilled water to make a 1-M stock solution. The ER antagonist ICI 182,780 (ICI; Sigma-Aldrich), the MAPK inhibitor PD98059 (PD; catalog no. 9900; Cell Signaling Technology), and the epidermal growth factor receptor (EGFR) inhibitor Tyrphostin AG 1478 (catalog no. 9842; Cell Signaling Technology) were dissolved in DMSO.

*Cell proliferation assay (MTS)*. CellTiter 96® Aqueous One Solution Cell Proliferation Assay (MTS, G3581; Promega) was used to measure cell proliferation according to the manufacturer’s instructions. Briefly, cells were seeded into 96-well plates and cultured in phenol red–free DMEM/F12 medium containing 10% charcoal/dextran-stripped fetal bovine serum (FBS) for 24 hr, followed by various Cd treatments without or with PD (10 μM) preincubation (2 hr).

*ER*α *and ER*β *competitive binding assay*. The binding affinity of E_2_ and Cd to ERα and ERβ was evaluated by fluorescence polarization following the instructions provided in the LanthaScreen® TR-FRET Competitive Binding Assays for ERα and ERβ (PV6041 and PV6042, respectively, Invitrogen). E_2_ or Cd, at increasing concentrations, was added to ER/Fluormone^TM^ ES2 Green mixer according to the manufacturer’s protocol.

*Transient transfection luciferase assays*. Cells were transfected using Lipofectamine® RNAiMAX Transfection Reagent (catalog no. 13778-075; Invitrogen by Life Technology) as described previously (Gao et al. 2012). Transfected cells, equipped with luciferase reporters, were maintained in phenol red–free medium containing 10% charcoal/dextran-stripped FBS for 24 hr prior to treatment with 10 nM E_2_ or Cd (0.01, 0.1, 1.0, 10, 20 μM) in the presence or absence of 1 μM ICI for 24 hr. Luciferase activity was measured using the Dual-Luciferase Reporter Assay System (Promega).

*Western blot analysis*. Whole cell lysates were obtained and used for Western blotting as described previously (Gao et al. 2012). The primary antibodies phospho-p44/42 MAPK (catalog no. 9101), p44/42 MAPK (catalog no. 9102), phospho-EGFR (catalog no. 3777), and EGFR (catalog no. 2232) (Cell Signaling Technology) were diluted at 1:1,000. We used ECL (enhanced chemiluminescence) horseradish peroxidase (HRP)-linked rabbit IgG (1:5,000, catalog no. NA934; GE Healthcare) the secondary antibody and HPRT (hypoxanthine-guanine phosphoribosyltransferase) antibody (sc-376559, Santa Cruz) as the loading control. The density of the respective bands was quantitated using a densitometer with AlphaView Software for FluorChem Systems (ProteinSimple™).

*Phosphorylation of receptor tyrosine kinases (RTKs).* To assess the phosphorylation status of human receptor tyrosine kinases (RTKs), we used Proteome Profiler™ Human Phospho-RTK Array Kits (catalog no. ARY001; R&D Systems) according to the manufacturer’s protocol. Briefly, aliquots of cell lysates were incubated with the RTK array membranes spotted with 42 anti-phospho-RTK antibodies. An HRP-conjugated pan anti-phospho-tyrosine antibody was then used to detect phosphorylated signals.

*Confocal immunofluorescence staining*. Pretreated cells were harvested, processed, and stained for confocal immunofluorescence microscopy as previously reported (Gao et al. 2012). Briefly, fixed cells were incubated with phospho-p44/42 MAPK antibody (1:100) at 4°C overnight, followed by incubation with Alexa Fluor® 594 goat anti-rabbit IgG secondary antibody (1:3,000, red fluorescence; catalog no. A11037; Molecular Probes) at room temperature for 1 hr. After counterstaining with DAPI (4´,6-diamidino-2-phenylindole; catalog no. D1306; Molecular Probes) for 30 min, slides were examined under a Zeiss LSM510-UV meta confocal microscope (Carl Zeiss).

*Statistical analysis*. All experiments were performed at least three times in duplicate. Results are expressed as mean ± SE. Cell proliferation data were analyzed by one-way analysis of variance (ANOVA) followed by Dunnett’s multiple comparisons test. Luciferase assay data and MTS data were analyzed by two-way ANOVA followed by Sidak’s multiple comparisons test. Two-tailed Student’s *t*-tests were used to compare pairs of time points for data on phosphorylation and RTK expression (SAS 9.3; SAS Institute Inc.). For binding assays, concentrations producing 50% of the maximum inhibition (IC_50_) were estimated from Hill models using Prism® 6.02 (GraphPad Software). *p*-Values < 0.05 were considered statistically significant.

## Results

**Figure 1:**
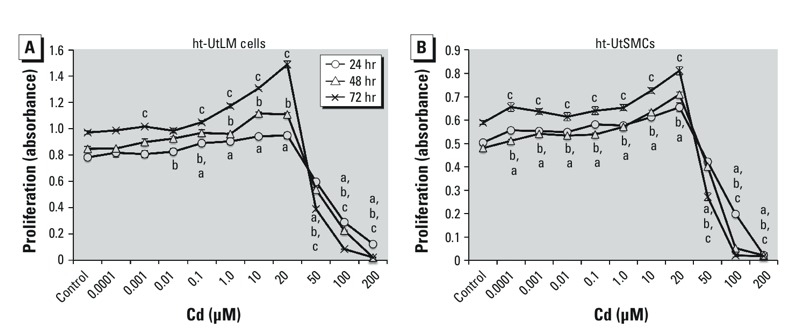
The effects of Cd on cell growth in ht-UtLM cells (*A*) and ht-UtSMCs (*B*) as measured by the MTS proliferation assay. Cd concentrations < 50 μM significantly increased cell growth in ht-UtSMCs, and at most low concentrations in UtLMs, whereas ≥ 50 μM Cd significantly inhibited cell growth at 24 hr, 48 hr, and 72 hr in both cell lines. Data were collected with an absorbance wavelength of 490 nm. Data represent mean ± SE of experiments repeated three times with independent cultures. ***^a^****p *< 0.05 compared with control at 24 hr. ***^b^****p *< 0.05 compared with control at 48 hr. ***^c^****p *< 0.05 compared with control at 72 hr.

*Effects of Cd on cell proliferation in ht-UtLM cells and ht-UtSMCs*. To evaluate the effects of Cd exposure on proliferation of human ht-UtLM cells and ht-UtSMCs, we conducted MTS proliferation assays using Cd at concentrations of 0.0001 μM to 200 μM. Compared with vehicle controls, ht-UtSMCs and ht-UtLM cells incubated with Cd for 24 hr, 48 hr, or 72 hr showed statistically significant proliferative responses as measured by absorbance at 490 nm, (Figure 1A,B). Therefore, we chose two representative intermediate doses (0.1 and 10 μM) to carry out further mechanistic studies.

*Influence of Cd on ER*α *or ER*β *responses* in vitro. We sought to determine whether ERs are involved in the proliferative effects observed in ht-UtLM cells and ht-UtSMCs after Cd exposure. First, we conducted competitive binding assays to examine the binding affinity of Cd to ERα and ERβ. The affinity of Cd to bind ERα or ERβ was nondetectable at concentrations ranging from 0.01 nM to 10 mM, whereas E_2_ bound to ERα and ERβ with high affinity (calculated IC_50_ of about 1.04 nM and 0.93 nM, respectively) (see Supplemental Material, Figure S1). Next, we determined whether Cd could modulate ER-dependent gene regulation in ht-UtLM cells and ht-UtSMCs. By using a luciferase reporter system, we found that 10 nM E_2_ resulted in significant responses in ERE-mediated luciferase activity in hERα and hERβ, which was fully abrogated by 1.0 μM ICI; however, Cd had a negligible influence on hERα or hERβ luciferase activity in ht-UtLM cells or ht-UtSMCs (see Supplemental Material, Figure S2). Collectively, these results do not support that Cd directly interacts with either ERα or ERβ *in vitro*. Therefore, we speculate that nonclassical ER mechanisms might be responsible for the proliferative effects observed in ht-UtLM cells and ht-UtSMCs after Cd treatment.

**Figure 2:**
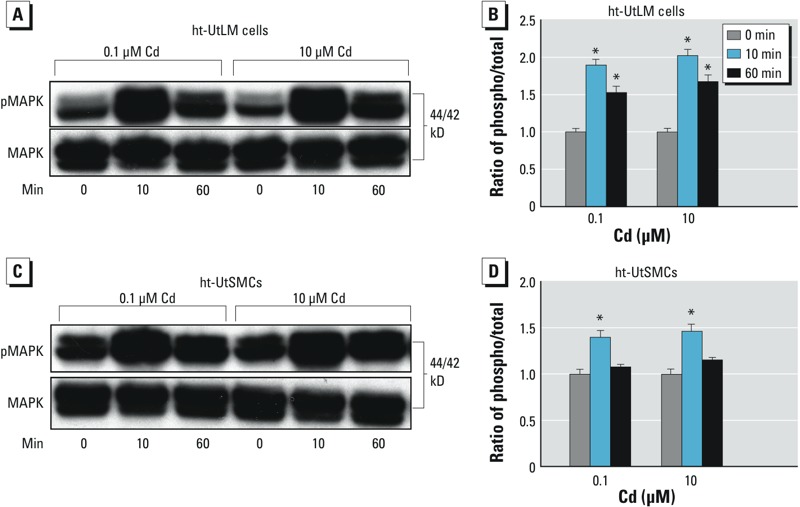
Effect of Cd (0.1 μM or 10 μM) on the phosphorylation (phospho) of p44/42 MAPK (pMAPK) in ht-UtLM cells (*A*,*B*) and ht-UtSMCs (*C*,*D*) shown by Western blots (*A,C*) and quantitation of the blots (*B,D*). Cd treatment resulted in increased phosphorylation of p44/42 MAPK at 10 min and 60 min. Quantitative data represent mean ± SE of experiments repeated three times with independent cultures. *p < 0.05 compared with the 0-min time point.

*p44/42 MAPK pathway and Cd-induced cell proliferation in ht-UtSMCs and ht-UtLM cells*. The MAPK pathway, well recognized as a critical mediator of cell proliferation in both normal growth and tumorigenic overgrowth, has been reported to be activated after exposure to Cd (Ali et al. 2010). Therefore, we evaluated the influence of Cd on activation of p44/42 MAPK in ht-UtLM cells and ht-UtSMCs. Using Western blotting, we found that treatment with 0.1 μM and 10 μM Cd resulted in marked increases in phosphorylation of p44/42 MAPK as early as 10 min in ht-UtLM cells (*p* < 0.01, vs. 0 min) (Figure 2A,B) and in ht-UtSMCs (*p* < 0.01, vs. 0 min) as well (Figure 2C,D). Our data indicate that the p44/42 MAPK pathway was activated by Cd and occurred as an early event in both ht-UtLM cells and ht-UtSMCs.

**Figure 3:**
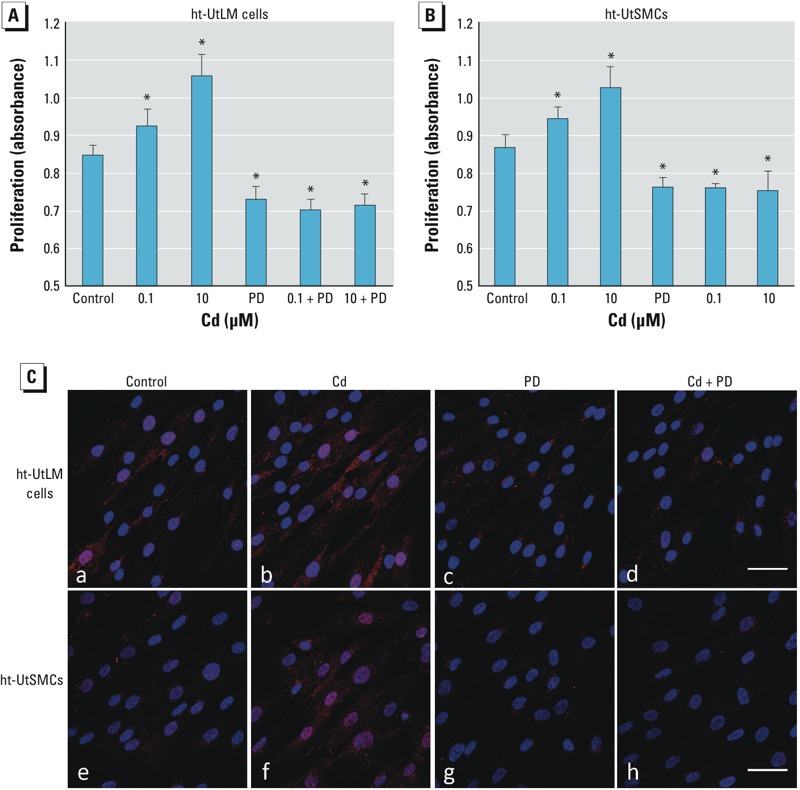
Effect of PD98059 (PD) on Cd-induced cell proliferation and p44/42 MAPK phosphorylation. Cell proliferation was evaluated in ht-UtLM cells (*A*) and ht-UtSMCs (*B*) treated with vehicle (control), Cd (0.1 μM or 10 μM), with 10 μM PD98059 (PD) alone, or Cd in combination with 10 μM PD for 72 hr. The experiments were repeated three times with independent cultures. Absorbance values were determined at a 490 nm wavelength. Data are presented as mean + SE (*n* = 6). (*C*) Confocal images of ht-UtLM cells (a,b,c,d) and ht-UtSMCs (e,f,g,h) treated with vehicle (control; a,e), 10 μM Cd (b,f), PD (50 μM; c,g), or Cd plus PD (d,h) for 10 min. Red indicates phospho-p44/42 MAPK, and blue indicates DAPI staining; bar = 50 μm. *p < 0.05 compared with control.

We evaluated whether the activation of the p44/42 MAPK pathway plays a role in Cd-induced cell proliferation. By adding a specific ERK inhibitor (PD, 10 μM) prior to Cd treatment (0.1 and 10 μM), Cd-induced cell proliferation was substantially abolished (*p* < 0.05, vs. Cd alone) in both cell types (Figure 3A,B). As shown in Figure 3C, treatment with 10 μM Cd resulted in robust activation of p44/42 MAPK as indicated by intense red positive signals in ht-UtLM cells and ht-UtSMCs (Figure 3C-b, 3C-f), whereas PD dramatically inhibited phospho-p44/42 MAPK expression (Figure 3C-c and 3C-g). Cd administration in the presence of PD did not result in activation of p44/42 MAPK (Figure 3C-d, 3C-h). Taken together, these data suggest that Cd-induced cell proliferation in ht-UtLM cells and ht-UtSMCs was mediated by activation of p44/42 MAPK.

**Figure 4:**
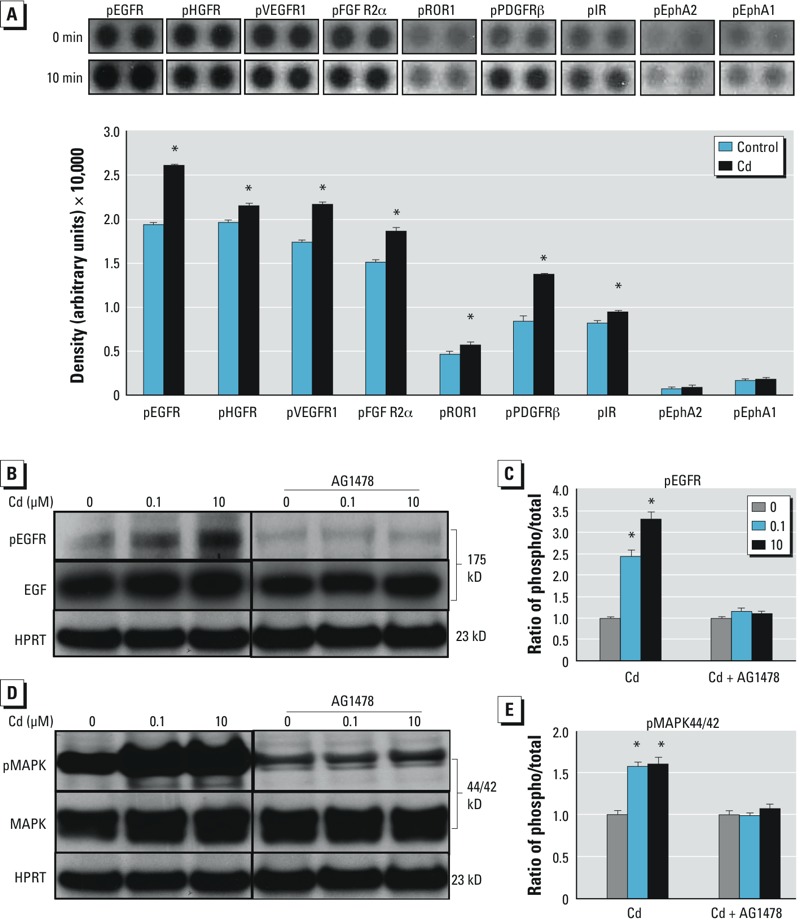
Effect of Cd on the phosphorylation (phospho) of receptor tyrosine kinases (RTKs) as well as EGFR (pEGFR) and p44/42 MAPK (pMAPK) activation in ht-UtLM cells. (*A*) Expression of growth factor RTKs in ht-UtLM cells after Cd (10 μM) treatment for 10 min shown by a representative RTK array (top) and as quantitated dot blot intensity values (mean ± SE) of ht-UtLM cells (bottom). The up‑regulated RTKs were EGFR, hepatocyte growth factor receptor (HGFR), vascular endothelial growth factor receptor (VEGFR1), fibroblast growth factor receptor 2 (FGFR2α), receptor tyrosine kinase-like orphan receptor 1 (ROR1), platelet-derived growth factor receptor beta (PDGFRβ), and insulin receptor (IR); the Ephrin (Eph) receptors EphA2 and EphA1 were not significantly expressed. The array was repeated at least three times. (*B–E*) Effect of 0.1 or 10 μM Cd on phosphorylation of EGFR (*B*,*C*) and p44/42 MAPK (*D*,*E*) at 10 min. In the presence of AG1478 (1 μM), an EGFR inhibitor, the increased EGFR phosphorylation induced by Cd was abolished; AG1478 also abrogated Cd-induced phosphorylation of p44/42 MAPK. The experiments were repeated three times with independent cultures. *p < 0.05 compared with 0 min.

*Cd-induced p44/42 MAPK phosphorylation is EGFR-dependent in ht-UtLM cells*. Various cell surface growth factor receptors (the RTKs) can trigger the p44/42 MAPK cascade and phosphorylation. In an attempt to identify specific upstream RTKs involved in Cd-induced p44/42 MAPK activation, we used phosphorylation RTK arrays on ht-UtLM cells and ht-UtSMCs incubated with 10 μM Cd for 10 min. Among 42 RTKs, seven candidate proteins were significantly expressed (*p* < 0.05, vs. 0 min) in ht-UtLM cells, with EGFR most highly expressed at baseline, and phosphorylation significantly increased at 10 min after Cd exposure (Figure 4A). The phosphorylated RTKs were differentially expressed in ht-UtSMCs compared to ht-UtLM cells following Cd treatment. In ht-UtSMCs, the most highly phosphorylated RTKs were Ephrin receptors, which maintain a critical role in angiogenesis (see Supplemental Material, Figure S3).

Because EGFR has recently been reported to mediate Cd-induced cell proliferation and survival (Carpenter and Jiang 2013; Martinez Flores et al. 2013), we sought to further determine the contribution of EGFR phosphorylation in p44/42 MAPK activation in the ht-UtLM cells. Cd treatment resulted in phosphorylation of EGFR, which was largely disrupted by the addition of AG1478 (1 μM), a selective EGFR-RTK inhibitor (Figure 4B,C). Accordingly, p44/42 MAPK activation induced by Cd was substantially abolished in the presence of AG1478 (Figure 4D,E). In short, these data suggest that Cd-induced p44/42 MAPK activation is EGFR dependent in ht-UtLM cells.

## Discussion

Cd is a heavy metal associated with ubiquitous air and water pollution that is also a contaminant in cigarette smoke. Circulating levels of Cd in chronically exposed women have been reported to be as high as 0.33–3.5 μg/L (Nasiadek et al. 2011). Moreover, Pollack et al. (2011) observed higher concentrations of Cd in tissue (0.047 and 0.075 μg Cd/g wet tissue in leiomyoma and myometrium, respectively) than in blood. In the present study, we found that environmentally relevant concentrations of Cd sufficiently induced cell proliferation in estrogen-responsive ht-UtLM cells and ht-UtSMCs. These effects were more likely to be mediated through activation of the p44/42 MAPK pathway than through direct interactions with ERα and ERβ. These data suggest that Cd should be considered an environmental risk factor for uterine fibroids and that EGFR could be a potential target in managing this risk. Our findings add benign tumors, such as uterine fibroids, to a long list of targets and adverse effects of Cd exposure.

The acute toxic effects of high concentrations of heavy metals such as lead and arsenic have long been acknowledged as life threatening. Moreover, recently, considerable efforts have been invested in exploring the adverse health effects of low-level and chronic exposures to heavy metals. For example, long-term, low-level lead exposures in children have been reported to lead to compromised neurobehavioral/cognitive capabilities (Olympio et al. 2009), and chronic Cd exposure has been associated with cancerous transformation of epithelial cells *in vitro* (Benbrahim-Tallaa et al. 2009; Jing et al. 2012). However, attempts to demonstrate the endocrine-disrupting or estrogenic effects of low-level Cd exposure in *in vitro* studies have produced inconsistent results (Höfer et al. 2010; Isidori et al. 2010; Silva et al. 2006). Nevertheless, these complexities coincidently support the notion that further investigations regarding the effects of Cd on human health, including the endocrine and reproductive systems, hold significant interest and urgency.

In regard to female reproductive health, we observed that Cd induced cell proliferation in ht-UtLM cells and ht-UtSMCs in a classical ER-independent manner. Previous studies have reported that Cd has estrogen-like activity and acts as an endocrine-disrupting chemical (Höfer et al. 2010; Kluxen et al. 2012). Environmentally relevant doses of Cd have been found to induce several estrogenic responses both in cultured breast cancer cell lines and in rats via the ER (Höfer et al. 2010; Kluxen et al. 2012; Siewit et al. 2010; Zang et al. 2009). In contrast, our data consistently showed that Cd did not directly bind to human ERα or ERβ and had no significant ER transcriptional activity in the presence or absence of ICI. These negative, but important, findings suggest that the effects of Cd on ht-UtLM cells and ht-UtSMCs most likely occur in a nonclassical ER manner, without significant contributions from ER binding or transactivation. Our findings are in agreement with those of Ali et al. (2010, 2012) who found that the estrogenic effects of Cd may be mediated, in part, by the MAPK/ERK1/2 signaling pathway. Ali et al. (2010, 2012) ruled out classical ER signaling through ERE-regulated genes in Cd-induced estrogenic responses observed *in vivo* and observed that activation of MAPK pathways was a mode of action for Cd. Liu et al. (2008) suggested that rapid activation of ERK1/2 and AKT in human breast cancer cell lines may occur through membrane ERα and GPR30, suggesting the presence of crosstalk between hormone and growth factor signaling pathways involved in Cd-induced cell signaling.

There are a number of factors that might account for these differences in observations regarding Cd’s estrogenicity. Variability and lack of standardized protocols for ER binding and transactivation assays make interlaboratory comparisons and validations difficult (Silva et al. 2006). In addition, differences in estrogenic responses observed with Cd treatment may be explained by variations in ER content, transcription factors, and coregulators present in diverse cell types utilized in *in vitro* studies. (Heldring et al. 2007; Wilson et al. 2004).

Another important finding in our study is that EGFR-dependent p44/42 MAPK activation appears to be critical in Cd-induced cell proliferation in ht-UtLM cells. MAPK pathways are evolutionarily conserved kinase modules that link extracellular signals to the machinery that controls fundamental cellular processes such as normal growth, proliferation, and differentiation (McKay and Morrison 2007). The ERK1/2 pathway, the most studied mammalian MAPK pathway, is dysregulated in approximately one-third of all human tumors including uterine fibroids. Activation of the RTK/MAPK pathway has been well documented in the development of uterine fibroids by our laboratory (Di et al. 2008; Yu et al. 2008) as well as by other investigators (Jiang et al. 2010). Interestingly, we found that EGFR phosphorylation was up-regulated by Cd, which mimicked the effects of E_2_; Shimomura et al. (1998) reported that E_2_ can up-regulate EGFR expression in cultured human uterine leiomyoma cells, and we found similar results in fibroid tissue samples from women in the proliferative (estrogenic) phase of the menstrual cycle (Yu et al. 2008). Other investigators have also found an association between Cd exposure and the induction of MAPK (Ali et al. 2010) and EGFR expression (Kundu et al. 2011).

Given that EGFR is a critical molecule linked with multiple human tumors, our findings may have many important clinical implications (Ciarmela et al. 2011). It is possible that Cd may have synergistic effects on uterine fibroids in settings in which Cd exposure occurs in combination with exposures to other EGFR-inducers/activators, such as estrogen. It is also promising that interventions targeting EGFR might be meaningful in managing the effects of Cd exposure on uterine fibroids and other disorders (Ciardiello and Tortora 2008; Paez et al. 2004). Besides these implications, there are also several other important directions that should be encouraged in this field. Because excessive extracellular matrix (ECM) is another critical feature of uterine fibroids, it may be extremely helpful to evaluate the full spectrum of risks of Cd on fibroids by exploring the potential effects of Cd on ECM turnover in ht-UtLM cells and ht-UtSMCs. In addition, optimized animal studies and human cohort studies may strengthen the viewpoint that Cd is an environmental estrogen mimic and a risk factor for uterine fibroids and other reproductive tract diseases.

## Conclusions

**Figure 5:**
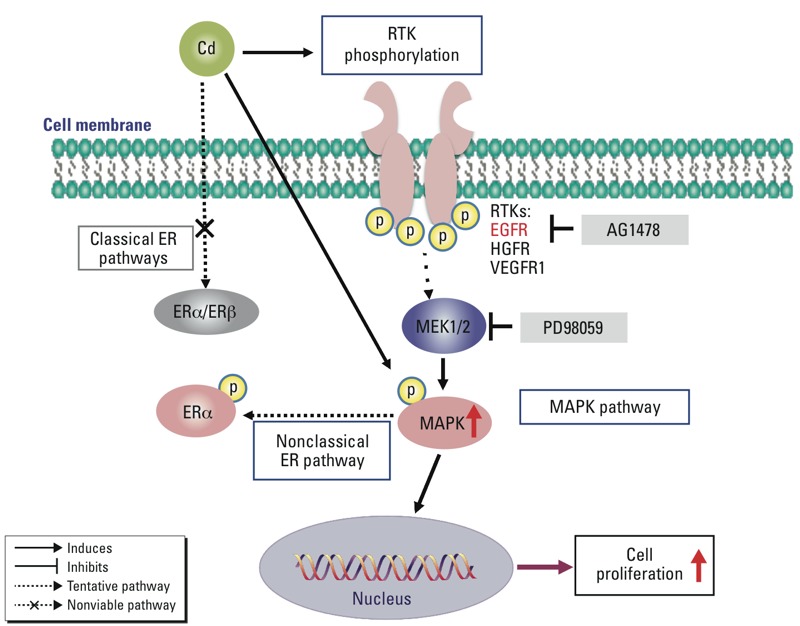
Schematic diagram of proposed molecular mechanism of EGFR and p44/42 MAPK (MAPK) phosphorylation in Cd-induced cell growth in fibroid cells. Classical ER pathways are not directly involved in Cd-induced cell proliferation in ht-UtLM cells. EGFR and MAPK played a role in Cd-induced proliferation in ht-UtLM cells. We propose that Cd mediates the phosphorylation of growth factor RTKs, such as EGFR, HGFR, and VEGFR1, which in turn activate downstream effector MAPK. The EGFR inhibitor AG1478 can diminish Cd-induced EGFR and MAPK phosphorylation; the MAPK inhibitor PD98059 can also decrease Cd-induced MAPK phosphorylation and cell growth.

In the present study we found that Cd-induced growth in ht-UtLM cells and ht-UtSMCs was not mediated by a classical ER mechanism of receptor binding and ERE-mediated gene activation, but through nongenomic pathways involving differential activation of growth factor receptors and subsequent MAPK/ERK1/2 phosphorylation (Figure 5). Our results suggest that Cd is a potential environmental risk factor for uterine fibroids. Further exploration of Cd-induced nongenomic signaling and the interaction between the different signaling pathways may be critical for developing new preventive strategies and risk assessment exposure paradigms for fibroids and other hormonally regulated disorders and cancers.

## Supplemental Material

(940 KB) PDF
