# Supplementary material for: Cadmium and Proliferation in Human Uterine Leiomyoma Cells: Evidence of a Role for EGFR/MAPK Pathways but Not Classical Estrogen Receptor Pathways
Source: Environ Health Perspect. 2014 Oct 24;123(4):331–6. doi: 10.1289/ehp.1408234 (PMC4384203; doi:10.1289/ehp.1408234)
Supplement: (940 KB) PDF [file ehp.1408234.s001.508.pdf]

Environmental Health Perspectives doi:10.1289/ehp.1408234

Supplemental Material

Cadmium and Proliferation in Human Uterine Leiomyoma Cells: Evidence of a Role for EGFR/MAPK Pathways but Not Classical Estrogen Receptor Pathways

Xiaohua Gao, Linda Yu, Alicia B. Moore, Grace E. Kissling, Michael P. Waalkes, and Darlene Dixon

A

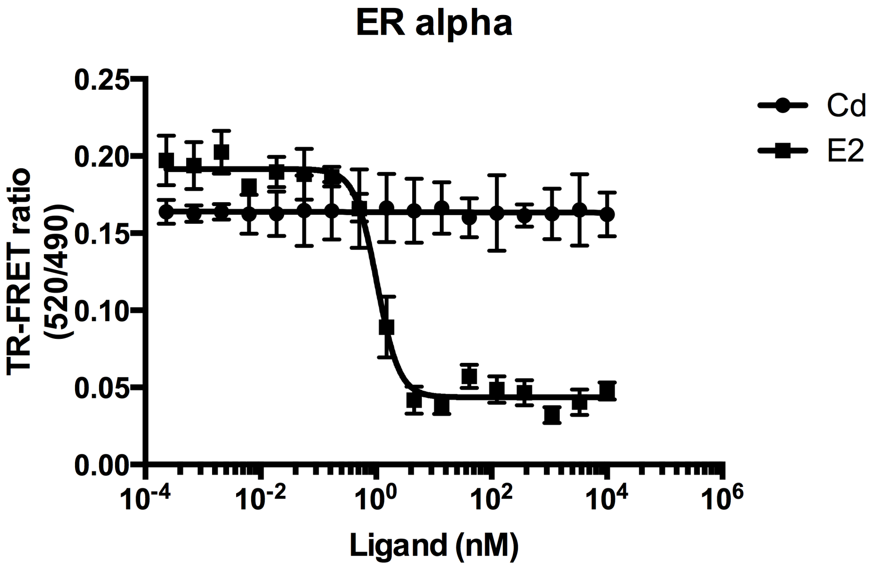

B

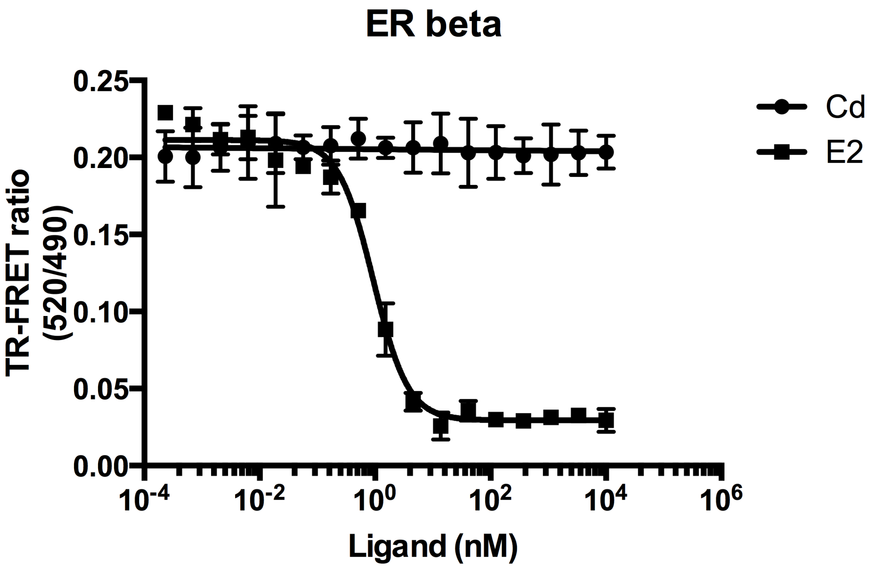

Figure S1. Cd does not bind to ERα or ERβ. After a 4 h incubation period, increasing concentrations of E_2 showed high binding affinity to ERα (A) and ERβ (B), while Cd was less likely to bind ERα (A) or ERβ (B).

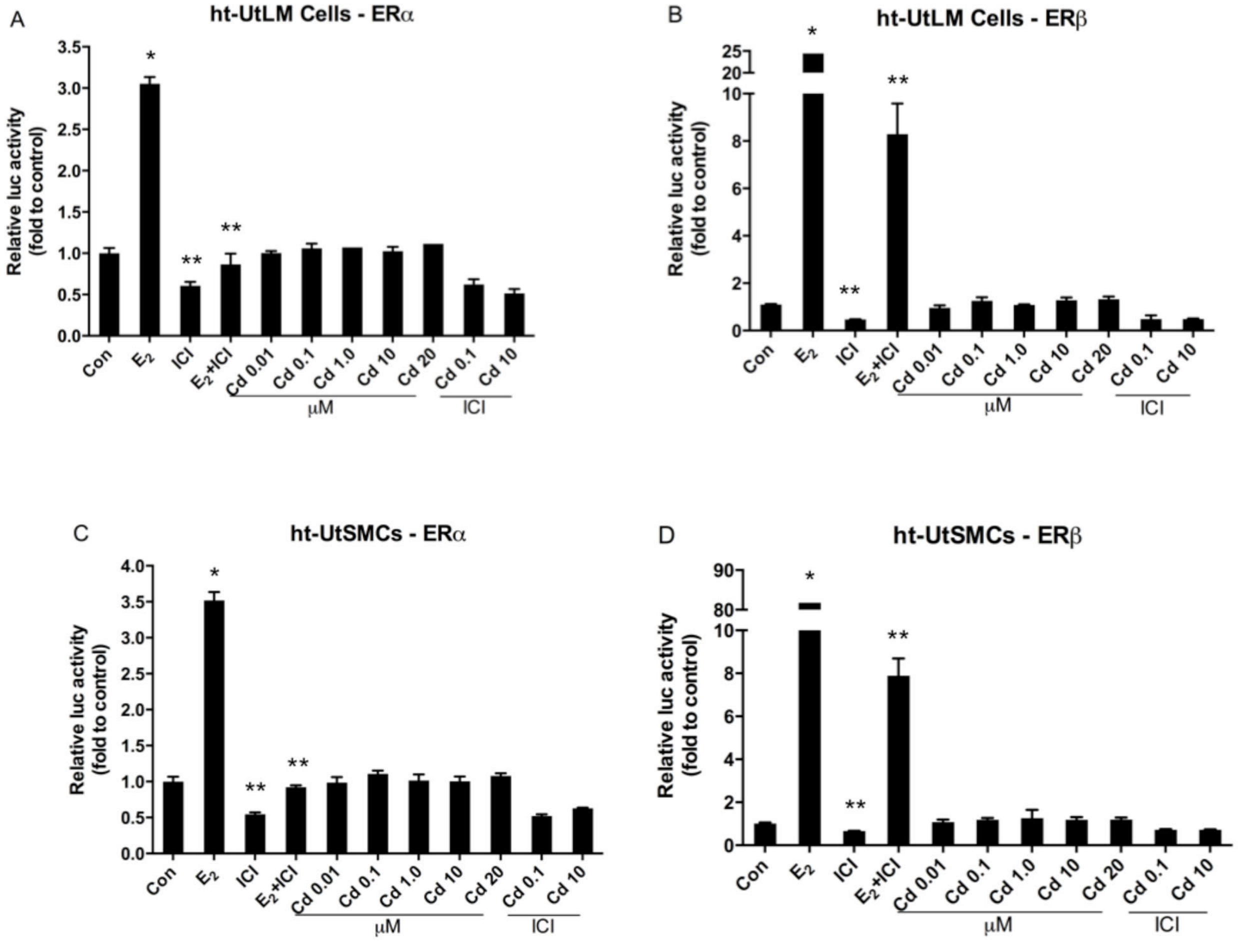

Figure S2. Transient transfection and luciferase assay in ht-UtLM cells and ht-UtSMCs. Relative luciferase activity in ht-UtLM cells and ht-UtSMCs transfected with hERα (A, C), hERβ (B, D), and 3×-Vit-ERE-TATA-Luc plasmids that were treated with DMSO (vehicle control, Con), 10 nM E_2, or 0.01, 0.1, 1.0, 10, 20 μM of Cd in the presence or absence of 1.0 μM ICI 182,780. *$p<0.05$ vs. Control=Con. **$p<0.05$ vs. E_2. The experiments were repeated three times with independent cultures.

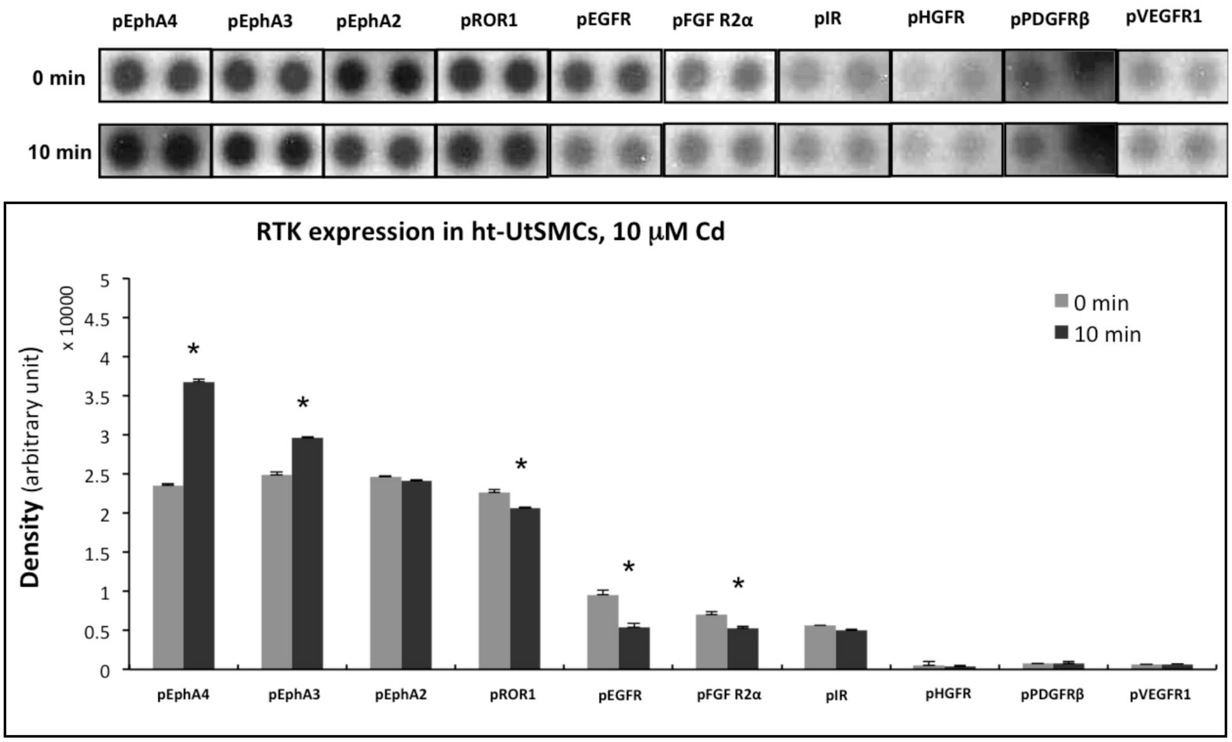

Figure S3. Phosphorylation (p) of growth factor Receptor Tyrosine Kinases (RTKs) in ht-SMCs. Growth factor RTKs were highly expressed after Cd (10 μM) treatment for 10 min in ht-SMCs. The significantly upregulated RTKs were Ephrin Receptor A 4 (EphA4), and Ephrin Receptor A 3 (EphA3). ROR (ROR1), EGF Receptor (EGFR), and FGF Receptor (FGFR2α) showed significantly decreased expression. While Ephrin Receptor A 2 (EphA2), Insulin Receptor (IR), HGF Receptor (HGFR), PDGF Receptor beta (PDGFRβ), and VEGF Receptor (VEGFR1) did not show significant changes. The array was repeated at least 3 times. The bars represent the dot blot intensity values for ht-UtSMCs. *$p<0.05$ vs. 0 min.
